# Analysis of the Anticancer Phytochemicals in *Andrographis paniculata* Nees. under Salinity Stress

**DOI:** 10.1155/2013/319047

**Published:** 2013-11-28

**Authors:** Daryush Talei, Alireza Valdiani, Mahmood Maziah, Sreenivasa Rao Sagineedu, Mohd Said Saad

**Affiliations:** ^1^Medicinal Plant Research Center, Shahed University, Tehran 33191 18651, Iran; ^2^Department of Cell and Molecular Biology, Faculty of Biotechnology and Biomolecular Sciences, Universiti Putra Malaysia (UPM), 43400 Serdang, Selangor, Malaysia; ^3^Department of Biochemistry, Faculty of Biotechnology and Biomolecular Sciences, Universiti Putra Malaysia (UPM), 43400 Serdang, Selangor, Malaysia; ^4^Department of Pharmaceutical Chemistry, School of Pharmacy, International Medical University, Bukit Jalil, 57000 Kuala Lumpur, Malaysia; ^5^Department of Crop Science, Faculty of Agriculture, Universiti Putra Malaysia (UPM), 43400 Serdang, Selangor, Malaysia

## Abstract

Salinity causes the adverse effects in all physiological processes of plants. The present study aimed to investigate the potential of salt stress to enhance the accumulation of the anticancer phytochemicals in *Andrographis paniculata* accessions. For this purpose, 70-day-old plants were grown in different salinity levels (0.18, 4, 8, 12, and 16 dSm^−1^) on sand medium. After inducing a period of 30-day salinity stress and before flowering, all plants were harvested and the data on morphological traits, proline content and the three anticancer phytochemicals, including andrographolide (AG), neoandrographolide (NAG), and 14-deoxy-11,12-didehydroandrographolide (DDAG), were measured. The results indicated that salinity had a significant effect on the aforementioned three anticancer phytochemicals. In addition, the salt tolerance index (STI) was significantly decreased, while, except for DDAG, the content of proline, the AG, and NAG was significantly increased (*P* ≤ 0.01). Furthermore, it was revealed that significant differences among accessions could happen based on the total dry weight, STI, AG, and NAG. Finally, we noticed that the salinity at 12 dSm^−1^ led to the maximum increase in the quantities of AG, NAG, and DDAG. In other words, under salinity stress, the tolerant accessions were capable of accumulating the higher amounts of proline, AG, and NAG than the sensitive accessions.

## 1. Introduction

Salinity is a major abiotic stress that causes important alterations in the plant growth and development. It may lead to the accumulation or reduction of certain metabolites [1–3]. As an aerial organ, leaves are directly exposed to various biotic and abiotic stresses, which demand metabolic adaptations for survival. Many of those metabolic changes such as sucrose, oligosaccharides, polyhydric alcohols, proline, and polyamines are observable in the leaves of plants exposed to abiotic stresses [4]. For example, the level of metabolites was increased in the leaves of salt-treated plants such as *Arabidopsis thaliana *[4], *Oryza sativa *[5], *Hordeum vulgare*, *Datura inoxia*, *Glycine max*, *Triticum aestivum *[5], and *Catharanthus roseus *[6]. Increases in certain amino acids (including proline), sugars (including sucrose, fructose, and glucose), and polyols (including inositols) have been reported in some plant species like grapevine [7], *Limonium latifolium *[8], and *Lotus japonicas *[9]. Wu et al. [10] identified Genistin and the group B saponins as the key secondary metabolites correlated with the salt tolerance in soybean varieties.

Since metabolic adaptations towards environmental stress including salinity affect global metabolic fluxes, some beneficial secondary metabolites of therapeutic importance (or so-called phytochemicals) would also be affected [11]. Plant species react against the environmental changes by producing some secondary metabolites such as soluble flavonoids, which protect the cellular structures from oxidative damage and osmotic stress [12]. In fact, a positive correlation between metabolite enhancement (flavonoid and proline) and relative water content (RWC) was reported in rice [13], reiterating the notion that metabolic adaptations are part of a complex mechanism to overcome threads from stresses. Therefore, the stress conditions have a strong impact on the responsible metabolic pathways for the accumulation of the related natural products [11]. However, improving the content of some active compounds such as flavonoids could be achieved under abiotic stress condition (as an agronomical approach) [12, 14] and biotechnological approaches (genetic transformation) [15] on a large scale in plants.

*Andrographis paniculata *Nees. is a medicinal herb from the family Acanthaceae that is blessed with bioactive compounds. The plant extract contains three major groups of phytochemical compounds, namely, diterpenes, flavonoids and stigmasterols [16]. Diterpene lactones are phytochemical structures, which could be found in different parts of this medicinal herb. Among these phytochemicals, three diterpene lactones consisting of andrographolide (AG), neoandrographolide (NAG), and 14-deoxy-11,12-didehydroandrographolide (DDAG) possess the highest bioactivity in treating the hardly curable diseases [17, 18]. The herb exhibited a wide scope of pharmaceutical properties such as anti-HIV [19], anti-H1N1 [20], anticancer [21], antihepatitis [22], anti-inflammatory [23], blood purifier, and antidiarrhea [24]. These compounds are produced mainly from aerial organs of the plant, especially leaves.

However, the production of the three phytochemicals in *A. paniculata* has been well studied under normal conditions, but there is a lack of concrete proof on the biosynthesis of these compounds under higher levels of salinity. It is assumed that the accumulation of these phytochemicals in *A. paniculata* (in the role of the secondary metabolites) enhances the capacity of the herb for salt tolerance. Therefore, the aim of this study was to determine the changes in the contents of the three main anticancer phytochemicals (AG, NAG, and DDAG) and their physiological implications in salt tolerance of *A. paniculata*. In the current study, phytochemical analysis was conducted on the four different accessions of *A. paniculata *under salinity stress. Here, we report how salt stress enhances the production of the key phytochemicals in the plant.

## 2. Results

### 2.1. Effect of Salinity Levels on Morphological Characters in *A. paniculata*

**Figure 1:**
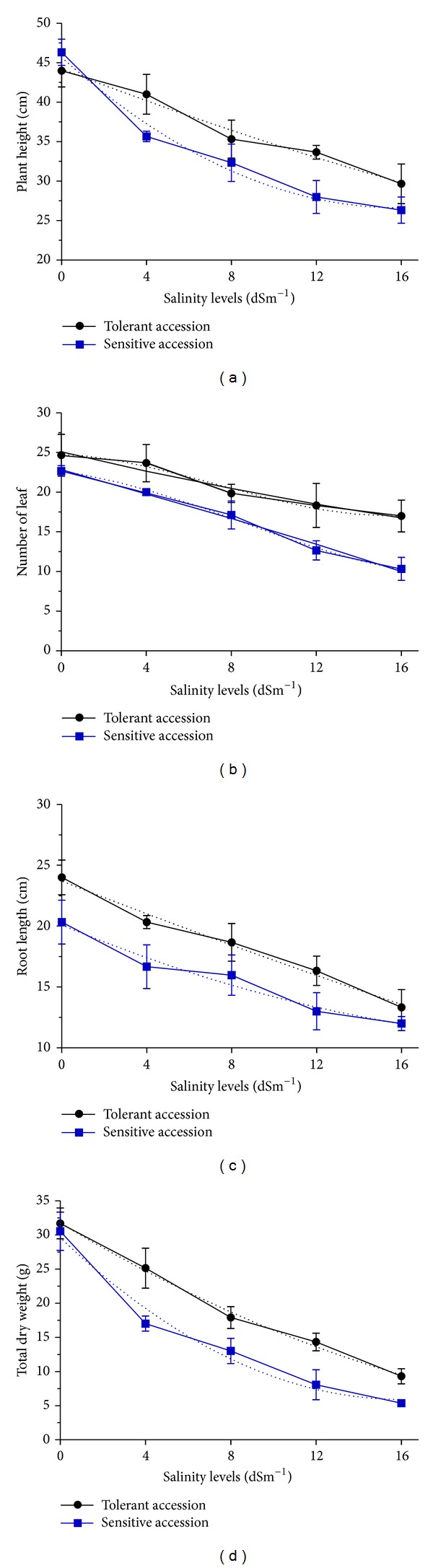
The effects of salinity levels on studied traits among the four accessions of *A. paniculata*. Increasing salinity levels led to decrease in PH (a), NL (b), RL (c), and TDW (d). Vertical bars represent standard error of mean for three samples.

The analysis of variance showed that salinity levels significantly affected NL, PH, RL and TDW. Variations due to salinity levels (SL) and accessions (AC) were highly significant (*P* ≤ 0.01). The interaction of SL × AC was not significant in terms of all studied traits (Table 1). The results showed that NL, PH, RL, and TDW were negatively correlated to the substrate concentration of NaCl after four weeks of salt stress (*P* ≤ 0.01). The reduction in sensitive accessions in terms of all studied morphological traits was higher than in tolerant accessions (Figure 1). The decreasing trend of all studied morphological traits was linear (Table 2). The dry weight was used to monitor the physiological response of each accession to salinity. After four weeks, a significant decrease in growth driven by salinity was apparent in all accessions. Twenty days after exposing the plants to 16 dSm^−1^ of salinity, the leaves started to fall. The 16 dSm^−1^ concentration of NaCl significantly reduced the dry weight by 75.65% compared to the control. An obvious reduction in TDW was seen in accession 11329 and this was taken as a symptom of salt sensitivity of this accession (Table 3). Accordingly, the growth of this accession (11329) was stopped after three weeks. By contrast, among the accessions, the highest TDW (21.03 g) was observed in accession 11264, whereas the lowest TDW (14.80 g) was recorded in accession 11329 (Figure 1).

### 2.2. Effect of Salinity Levels on Proline and the Three Anticancer Phytochemicals in *A. paniculata*

**Figure 2:**
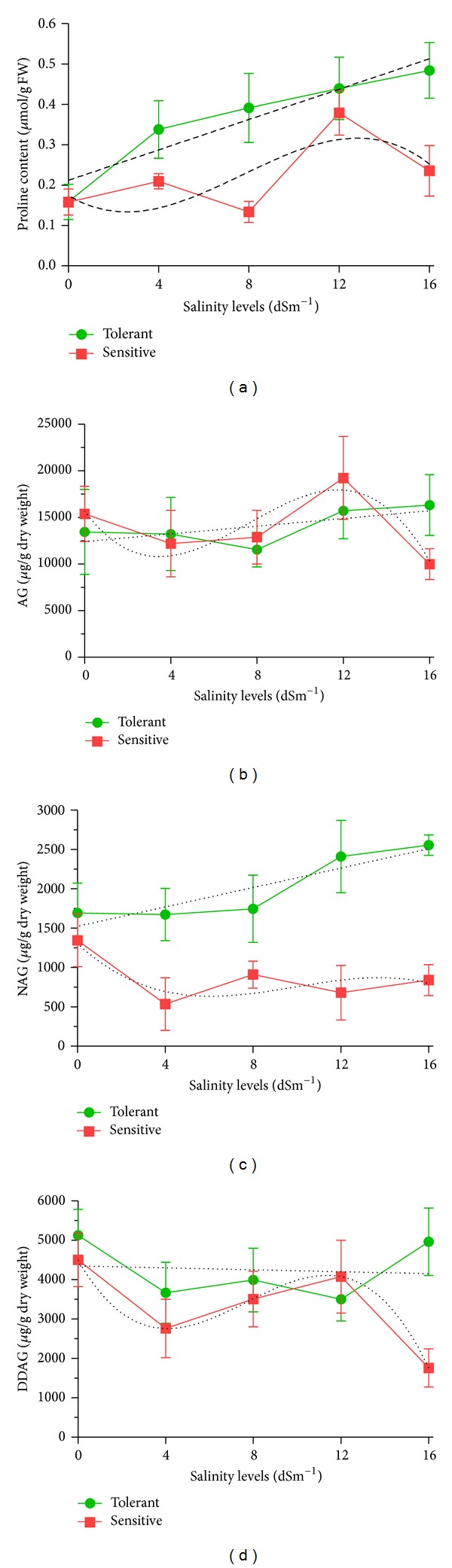
The effects of salinity levels on the measured physiological and phytochemical traits among tolerant and sensitive accessions of *A. paniculata*. Increasing salinity levels led to increase in proline (a), AG (b), and NAG (c) and decrease in DDAG (d). The trend of proline and the three main phytochemicals in tolerant accession were linear and positive, while in sensitive accession they were nonlinear. Vertical bars represent standard error of mean for three samples.

The analysis of variance showed that different levels of salinity affected the proline and the three main phytochemicals of *A. paniculata* significantly (*P* ≤ 0.05) (Table 1). Variation due to salinity levels (SL) was significant in terms of proline, AG, and NAG (*P* ≤ 0.05), while there were no significant differences among accessions based on DDAG under salt stress conditions. Seemingly, the accession-based variation followed the same trend as the SL's except for NAG (*P* ≤ 0.01) and proline (nonsignificant). The interaction of SL × AC was not significant for none of the aforementioned phytochemicals though (Table 1). The results showed that proline, AG, and NAG contents were positively correlated to the substrate concentration of NaCl (*P* ≤ 0.01), while the total crude extract amount was negatively correlated to salinity levels (Figure 2). The tolerant plants at a high salinity level (16 dSm^−1^) reached relatively higher proline, AG, and NAG content. However, the DDAG amount was reduced at higher levels of salinity (Figures 1 and 2). The increase in AG and NAG contents at high salinity level (16 dSm^−1^) was 177.57% and 131.18% in comparison to the control, respectively.

**Figure 3:**
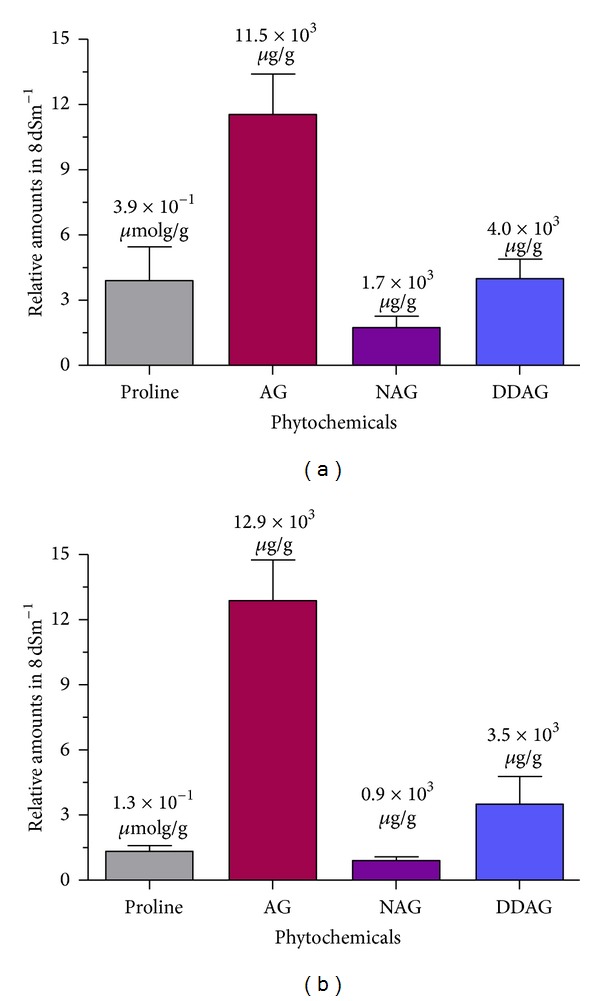
The relative amounts of phytochemicals in salt-tolerant (a) and salt-sensitive (b) accessions of *A. paniculata* when the plants' growth is inhibited up to 50% in 8 dSm^−1^. Vertical bars represent standard error of mean for each phytochemical.

Analysis of variance showed high significant differences (*P* ≤ 0.01) among the accessions for AG and NAG contents. The concentration of the AG, the most important cytotoxic principle in *A. paniculata* varied from 13940.7 *μ*g/g dry weight (11329) to 20392.7 *μ*g/g dry weight (11264), with a mean value of 16119.2 *μ*g/g dry weight (Table 3). The levels of NAG and DDAG in the dry matter ranged from 862.7 to 1195.3 *μ*g/g dry weight and from 3320 to 4618 *μ*g/g dry weight, respectively. Among the accessions, the highest NAG (2016 *μ*g/g dry weight) and DDAG (4618 *μ*g/g dry weight) were observed in accessions 11179 and 11264, respectively, whereas the lowest (862.7 and 3320 *μ*g/g dry weight) under the same condition belonged to accession 11329 (Table 3).  Figure 3 is a typical presentation of relative amounts for each phytochemical of interest when the plant growth was inhibited down to 50%.

The broad-sense heritability (*h*_*b*_^2^) was calculated for all studied characteristics. The heritability of proline was the highest (0.896 *≈* 0.90%); in contrast, the DDAG content gained the lowest heritability (0.66%) (Table 4). Heritability estimates are normally categorized as low if values are lower than 20%, moderate if the estimates ranged between 20 and 50%, and high if values were larger than 50% [25]. Therefore, proline, TDW, and NAG as the three highly heritable traits in* A. paniculata* are considered as direct criteria for assessing the response of the plant to salinity stress. In another word, the role of genetics (including all the gene effects such as additive, dominant, and epistatic) in differences of TDW, proline, TCE, AG, NAG, DDAG among the studied accessions is 89, 90, 81, 71, 87 and 66%, respectively. This can be utilized as a potential in the next breeding programs to develop the salt-tolerant varieties in *A. paniculata*.

**Figure 4:**
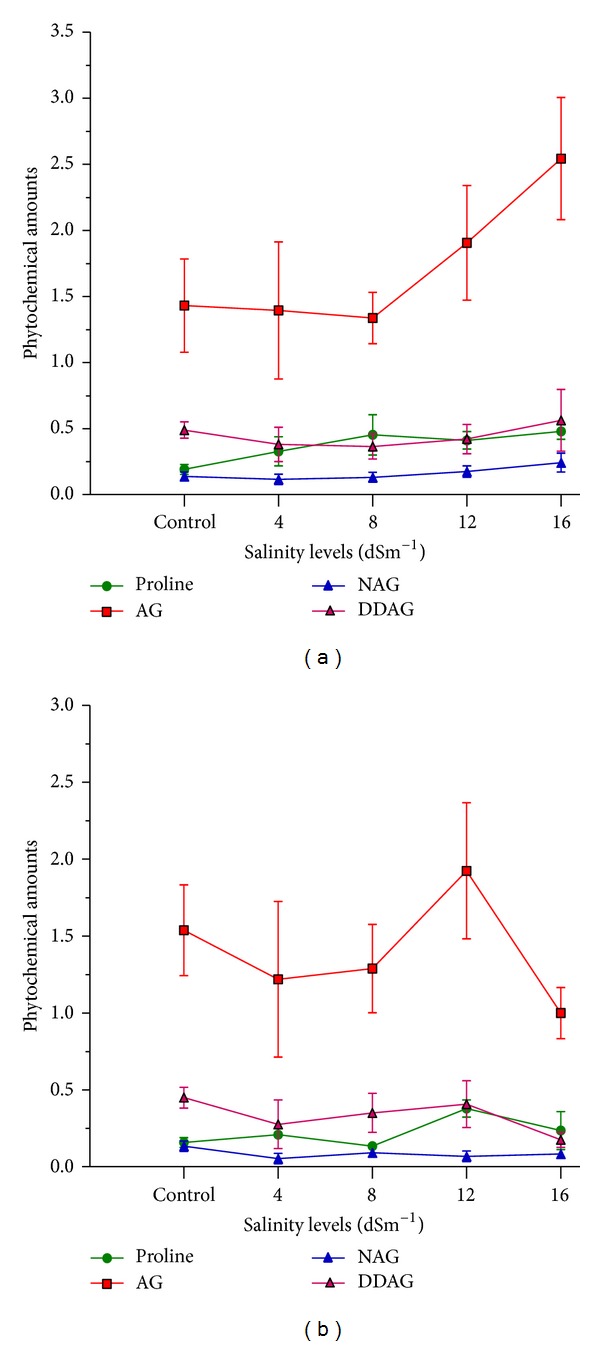
The correlative aspects of phytochemical production as well as the accumulation of proline in salt-tolerant (a) and salt-sensitive (b) *A. paniculata* accessions. Vertical bars represent standard error of mean for each phytochemical.

**Figure 5:**
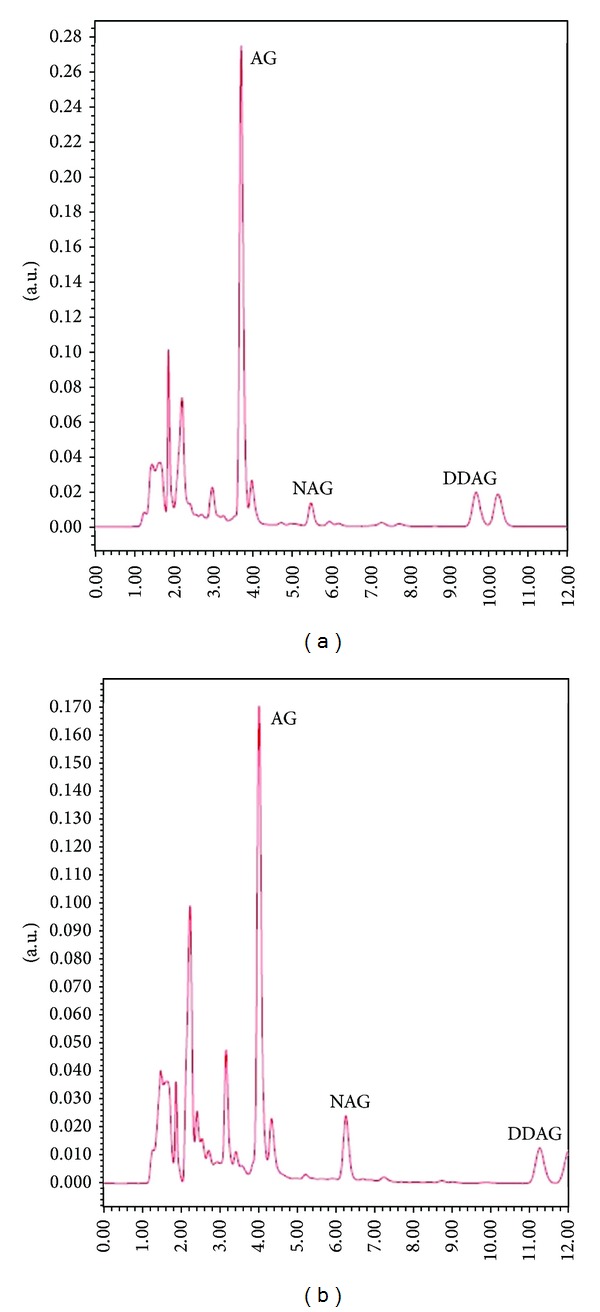
Chromatograms of the three phytochemicals in control (a) and 16 dSm^−1^ (b) treatments. The slight differences in retention times (RT) of the phytochemicals are due to the running of the samples in different batches using a refreshed mobile phase. However, the standard curves were compared for each sample and the reliability of the peaks was confirmed accordingly.

There were strong relationships between most studied traits. Interestingly, the correlation between proline as well as NAG with the other measured morphological characteristics was significant and positive in the salt-stressed plants. On the other hand, there was no significant correlation between the three anticancer phytochemicals and the other studied morphological traits, while correlations between AG, NAG, and DDAG were highly significant and positive (Table 5). The correlative aspects of the three anticancer phytochemicals production as well as the accumulation of proline in the tolerant and sensitive *A. paniculata* accessions have been presented in Figure 4. The presented chromatograms for the three phytochemicals reveal the related peaks of AG, NAG, and DDAG in control and 16 dSm^−1^ treatments (Figure 5).

## 3. Discussion

Accession 11329 showed a growth reduction of dry weight up to 82.4% (compared to 58.6% in 11264). By this time, the tolerant accession (11264) also had significantly higher proline and phytochemical contents and less leaf necrosis than sensitive accession (11329). Previous studies have demonstrated that the ability of *A. paniculata* to tolerate salt stress condition is associated with higher proline, K^+^, and K^+^/Na^+^ ratio and lower Na^+^ content [26].

The plants grown under salinity conditions performed different responses after four weeks of salt treatment. Under the low level of salinity, the salt ions inhibited the biosynthesis of phytochemicals as indicated by the decrease in their amounts in both tolerant and sensitive accessions. Surprisingly, under the extreme levels of salinity (12 and 16 dSm^−1^), the production of AG, NAG, and DDAG was increased in the tolerant accession. Moreover, the increase in proline content as well as in the three main phytochemical contents was observed maximum up to 12 dSm^−1^ salinity level, which has been reported as the high-threshold point of salt tolerance in this plant [26]. There is a possibility that at the low levels of salinity, the plants might be in the adaptive process against salinity stress due to the osmotic reduction of water surrounding the root system. We noticed that the major difference among the tolerant and sensitive accessions was related to their ability in tolerating high salt stress. At high salinity level, the tolerant plants were able to tolerate the salinity stress due to the accumulation of osmoregulation and compatible solutes such as proline and soluble flavonoids, while the sensitive accessions were unable to withstand the salinity stress due to the accumulation of sodium ion.

Generally, plants produce secondary metabolites in nature as defense mechanisms under different environmental stresses [27]. In this regard, our results indicated that proline as well as the AG and NAG contents is positively correlated with the substrate concentration of salt, in which the tolerant accessions were higher in proline, AG, and NAG contents compared with the sensitive ones. However, we are totally aware that not any correlation does imply causation, yet we believe that in this particular case and according to the previous experiences, our observations are in agreement with the concept of a close relationship between the availability of the secondary metabolites and self-defense systems of the plant species against abiotic stresses. Likewise, many researchers suggested that proline and soluble flavonoids are involved in osmotic adjustment, protecting the cellular structures from oxidative damage and osmotic stress by accumulation in the vacuole, and so play an important role in increasing the oxidative stress tolerance through protecting the chloroplast and photosynthetic systems versus solar radiation by absorbing UV [28, 29]. Our results indicated that proline content in the salt-stressed plants was peaked when the level reached 16 dSm^−1^. Consequently, the results suggested that increasing the biosynthesis of proline in the plant could protect the cellular structures from oxidative damage and osmotic stress. This event complies with the hypothesis that the *A. paniculata* accessions may operate different cellular mechanisms in response to high levels of Na^+^ in their tissues, so that the tolerant accessions tended to produce high amounts of proline and the three anticancer phytochemicals.

Reportedly, improving the content of some phytochemicals such as flavonoids as one of the most important pharmaceutical contents could be enhanced through salinity and drought stresses [12, 14, 15]. In accordance with other researchers' findings, who noticed a significant increase in polyphenolic compounds in *Zea mays *[30], flavonoids in *Hordeum vulgare *[31], phenolic contents in *Cuminum cyminum *[32], menthone in *Mentha pulegium *[33], and alkaloid content in *Catharanthus roseus *[6], our study indicated that certain levels of salinity could lead to a significant increase in contents of AG and NAG. As a matter of fact, a part of this enhancement could be due to an obvious reduction of the plant biomass. Enhancement of phenolic and flavonoid compounds in onion plant under salinity stress has been reported to improve the deleterious effect of salinity stress [34].

Accumulation of compatible solutes is nontoxic and does not disturb cellular functions even when they are present in high concentrations. These compounds in the cytoplasm can contribute to reduce the water potential in the cytoplasm by balancing the decreased water potential associated with Na^+^ accumulation in the vacuoles and the extracellular volume. These neutral organic compounds can also improve the inhibitory effects of high ion concentrations on enzymatic activity without interfering with protein structure and function [35].

Our results indicated a positive correlation between the three anticancer phytochemicals and proline accumulation, which might be closely related to tolerance abilities indicated by physiological performances. This finding matched up with the Chutipaijit et al. [13] outcomes who detected a positive correlation between flavonoid and proline accumulations and relative water content (RWC) in rice (*Oryza sativa* L. spp. Indicia).

To the best of our reference, the only conducted research related to salt stress in *A. paniculata *(prior to our studies) that could be compared with our results belongs to Rajpar et al. [36]. However, beside the different accessions used in their experiment (two accessions consisting of 11261 and 11265), there is another basic difference between our study and their experiment, whereas the highest salinity used by them never exceeded 5 dSm^−1^. Focusing on the result of the mentioned research reveals some interesting points, in which one of the used accessions (11265) behaved almost the same as the tolerant accessions of the present study (11179 and 11264), meaning that increasing the salinity up to 5 dSm^−1^, led to an increase in the contents of AG and NAG, while it caused a decrease in DDAG content. Unlike the accession 11265, the contents of AG, NAG, and DDAG in accession 11261 were all decreased with increasing salinity. In spite of the mentioned trends, the differences between those two accessions were not statistically significant [36]. Perhaps, the most important point that should be taken from the previous study is that 5 dSm^−1^ of salinity is not enough to evaluate the plant's real capacity in tolerating salt stress in terms of increasing AG, NAG, and DDAG contents. To be clearer, it seems that the accessions with high contents of the anticancer phytochemicals under normal condition would deal more successfully with salinity, as well.

## 4. Conclusion

However, the use of salinity to enhance the biosynthesis of the phytochemicals must be prudentially regarded, and it should not be forgotten that the increased concentration of bioactive compounds such as AG, NAG, and DDAG by salt stress in general is associated with a reduction of biomass production. In other words, the increase of these phytochemicals under salinity condition would be partially compensated by a decline in total biomass. However, this should not be interpreted in the way that saline water and soil are suggested as an alternative solution to increase the production of *A. paniculata*, but our outcomes clarified that the hydroponic culture and also running the cultivation of this herb in saline area could be considered as an affordable agricultural option. Fundamentally, it can be concluded that if the production of anticancer phytochemicals of *A. paniculata* (AG, NAG, and DDAG) is the central target of an agricultural system, then high salinity levels up to 16 dSm^−1^ should not be taken as a serious barrier, but if the total biomass is regarded as the main reason of development of this plant (such as forage production), then salinity would entirely be an obstacle to this end.

## 5. Materials and Methods

### 5.1. Chemicals

Solvents (AR and chromatography grade) for isolation and purification of the compounds were used as supplied by Fisher Scientific (Leicestershire, UK).

### 5.2. Plant Material and Growth Conditions

According to Talei et al. [37], two salt-tolerant (accessions 11179 and 11264) and two salt-sensitive accessions (accessions 11266 and 11329) of *A. paniculata* were collected from Agro Gene Bank, Universiti Putra Malaysia (Table 6). The seeds were germinated as described by Talei et al. [38] and then incubated under controlled growth chamber (light/dark regime of 14/10 h at 28–30°C, relative humidity 60–75%). The germinated seeds at two initial leaf stages were transferred into the Jiffy media. The 40-day seedlings were transferred from jiffy media into the pot with sand medium. After 30 days of culturing (almost in 70 days old), the plants were placed in different salinity levels.

### 5.3. Experimental Technique

The experiment was carried out with a split plot based on a randomized complete block design (RCBD) with two factors and three replicates. The factors were five different concentrations of saline water (control, 4, 8, 12, and 16 dSm^−1^) in main plots and four different accessions in submain plots. These salinities were applied using 41.1, 92.4, 143.7, and 193.4 mM of NaCl solution (calculated by fitting regression formula using different concentrations of NaCl). Since the highest amount of the active components is found just before the plant blooms [39], the 70-day-old plants were subjected to different salinity levels. Each plant was irrigated once a day with five levels of saline water. After every three salinity applications, plants were again irrigated with normal Hoagland nutrient solution. After a 30-day salinity exposure and before flowering, all plants were harvested and data on morphological traits (NL, PH, RL, and TDW), proline content, and the three main phytochemicals (AG, NAG, and DDAG) were measured.

### 5.4. Crude Extraction Methods

Arial parts of the plants were dried in the universal ventilated-electric oven (Memmert, Germany) at 55°C for 72 hours. The dried materials were chopped into small pieces and ground into fine powder form and were then extracted with a mixture of dichloromethane and methanol (DCM : ME) at the ratio of 1 : 1 [18].

A total of 50 g dried material of *A. paniculata* was used for extraction solvent with three replicates. The materials were soaked for three days at room temperature. The process was repeated several times with the same solvent system until the solvent portion becomes colorless. Whatman filter paper no. 1 was used for filtration of the solvent extracts. The solvent extract was concentrated under reduced pressure using a rotary evaporator. The concentrated extract was transferred into conical flasks and the remaining solvent was removed. A final drying was done by placing the concentrated extract in an electric oven at room temperature. The final dried extracts were measured and then replaced into small glass containers and after sealing were stored at −20°C for future analysis.

### 5.5. Preparation of Samples

Crude extract of each sample was dissolved as 1 mg/mL stock solution in HPLC grade methanol (Merck, Germany). One mL of each sample was finally filtered into HPLC vials using disposable polypropylene syringe filters with 0.2 *μ*m pore size and was then subjected to HPLC analysis [40, 41].

### 5.6. Preparation of Standards

AG (Sigma-Aldrich, USA, purity 98%), DDAG and NAG obtained from in-house standards collection were used as standard samples [41]. Stock solutions of standard AG, NAG, and DDAG (1 mg/mL) were dissolved in HPLC grade methanol. The stock solution was diluted with the same solvent to produce different concentration of working standards (0.01, 0.1, 0.2, 0.5, 1, 5, 10, 100, and 1000 *μ*g/mL). One mL of each standard sample was finally filtered into HPLC vials using the same syringe filters in five replicates [40, 41].

### 5.7. HPLC Analysis

Twenty *μ*L from each working standard solution in three replicates was injected into the HPLC. Calibration curve was generated by linear regression based on peak areas [42]. HPLC system was operated by Waters comprising Waters 600 Controller pumps and Waters 717 plus Auto sampler injector with a capacity of 96 samples. LiChrocart HPLC-Cartridge RP-18e 5 m (150 × 4.6 mm, Merck, Germany) was used as the stationary phase. The isocratic mobile phase was prepared with acetonitrile water (40 : 60 v/v), 0.1% (v/v) analytical grade orthophosphoric acid, a flow rate of 1 mL/min [40–42]. Detection was at 223 nm using Waters 486 Tunable Absorbance Detector (photodiode array detector).

## 6. Statistical Analysis

The SAS program version 9 was used for all statistical analyses including the raw data normality test and the main data analysis as well as for the Duncan's multiple range test (*P* ≤ 0.01). The Graphpad Prism software version 5 was used for drawing the graphs.

## Figures and Tables

**Table 1:** Variance analysis of salinity effects on morphological traits, proline, and the three main phytochemicals in *A. paniculata* accessions*. *

Source	df	Mean square								
		PH	NL	RL	TDW	Proline	TCE	AG	NAG	DDAG
SL	4	607.52**	190.44**	146.44**	1103.29**	0.093*	19.77**	78.60 × 10^6∗^	1.04 × 10^6∗^	2.99 × 10^6ns^
AC	3	41.98**	48.73**	27.44*	119.27**	0.052^ns^	1.63*	136.40 × 10^6∗^	4.00 × 10^6∗∗^	4.64 × 10^6ns^
SL × AC	12	13.95^ns^	6.94^ns^	1.93^ns^	9.23^ns^	0.020^ns^	0.47^ns^	66.94 × 10^6ns^	0.43 × 10^6ns^	2.23 × 10^6ns^
Error	30	9.57	8.87	6.67	14.42	0.036	0.39	55.18 × 10^6^	0.63 × 10^6^	2.42 × 10^6^

SL: salinity level, AC: accession, PH: plant height (cm), NL: number of leaf, RL: root length (cm), TDW: total dry weight (g), proline (*μ*mol/g FW), TCE: total crude extract (g), AG: andrographolide (*μ*g/g dry weight), NAG: neoandrographolide (*μ*g/g dry weight), and DDAG: 14-deoxy-11,12-didehydroandrographolide (*μ*g/g dry weight). Statistical significance is indicated by **(*P* ≤ 0.01), *(*P* ≤ 0.05), and ns (no significant).

**Table 2:** The comparison of decrease in studied morphological traits in tolerant and sensitive accessions of *A. paniculata* under salinity stress.

Accession	Characters			
	PH	NL	RL	TDW
Tolerant	−3.6	−2.1	−2.3	−5.6
Sensitive	−4.7	−3.2	−2	−5.9

PH: plant height (cm), NL: number of leaf, RL: root length (cm), and TDW: total dry weight (g).

**Table 3:** Effects of salinity levels on TDW, proline and three main phytochemicals in tolerant and sensitive accession of *A. paniculata* (Mean values ± standard error).

Accession	Salinity	TDW	Proline	AND	NAG	DDAG
Tolerant (11179)	1	36.95 ± 2.71	0.16 ± 0.04	13453.3 ± 4550.7	1693.3 ± 479.9	5126.7 ± 754.4
	2	21.52 ± 4.63	0.34 ± 0.13	13220.0 ± 3925.9	1673.3 ± 632.5	3666.7 ± 875.6
	3	17.45 ± 3.49	0.39 ± 0.15	11546.7 ± 1856.4	1746.7 ± 527.2	3990.0 ± 907.5
	4	13.17 ± 0.75	0.44 ± 0.08	15713.3 ± 2997.1	2410.0 ± 759.6	3503.3 ± 652.5
	5	9.45 ± 1.45	0.48 ± 0.07	16333.3 ± 3265.0	2556.7 ± 129.9	4963.3 ± 1055.5

Mean		19.71 ± 2.78	0.36 ± 0.05	14053.3 ± 1382.1	2016.0 ± 231.3	4250.0 ± 371.1

Sensitive (11329)	1	30.53 ± 3.57	0.16 ± 0.03	15380.0 ± 2954.7	1346.7 ± 335.8	4500.0 ± 676.8
	2	17.02 ± 1.10	0.21 ± 0.02	12196.7 ± 7553.4	536.7 ± 335.3	2760.0 ± 1584.1
	3	13.01 ± 1.84	0.13 ± 0.03	12886.7 ± 2872.4	910.0 ± 172.1	3506.7 ± 1270.6
	4	8.07 ± 2.20	0.38 ± 0.06	19246.7 ± 6442.4	680.0 ± 354.9	4076.7 ± 1524.5
	5	5.37 ± 0.33	0.24 ± 0.12	9993.7 ± 1656.4	840.0 ± 196.5	1756.7 ± 486.7

Mean		14.80 ± 2.49	0.22 ± 0.03	13940.7 ± 2023.2	862.7 ± 131.1	3320.0 ± 521.6

TDW: total dry weight (g), Proline (*μ*mol/g FW), TCE: total crude extract (g), AG: andrographolide (*μ*g/g dry weight), NAG: neoandrographolide (*μ*g/g dry weight), DDAG: 14-deoxy-11,12-didehydroandrographolide (*μ*g/g dry weight).

**Table 4:** Components of variance and broad-sense heritability of studied characters in *A. paniculata* accessions.

Components	Characters								
	PH	NL	RL	TDW	Proline	TCE	AG	NAG	DDAG
σ_*G*_^2^	41.98	48.73	27.44	119.27	0.139	1.63	1.36	0.04	0.046
σ_*P*_^2^	51.55	57.6	34.11	133.69	0.155	2.02	1.91	0.046	0.07
*h*_*b*_^2^	0.81	0.85	0.80	0.89	0.90	0.81	0.71	0.87	0.66

σ_*G*_^2^: genetic variance, σ_*P*_^2^: phenotypic variance, and *h*_*b*_^2^: broad-sense heritability. PH: plant height (cm), NL: number of leaf, RL: root length (cm), TDW: total dry weight (g), proline (*μ*mol/g FW), TCE: total crude extract (g), AG: andrographolide (*μ*g/g dry weight), NAG: neoandrographolide (*μ*g/g dry weight), and DDAG: 14-deoxy-11,12-didehydroandrographolide (*μ*g/g dry weight).

**Table 5:** Phenotypic correlation coefficient (*r*) among morphological and phytochemical traits in *A. paniculata* accessions under salt stress conditions.

	PH	NL	RL	TDW	Proline	TCE	AG	NAG	DDAG
PH	1								
NL	0.699**	1							
RL	0.708**	0.623**	1						
TDW	0.820**	0.688**	0.762**	1					
Proline	−0.091	−0.036	−0.089	−0.172	1				
TCE	0.885**	0.718**	0.687**	0.777**	−0.096	1			
AG	−0.145	−0.216	0.027	−0.109	0.243	−0.092	1		
NAG	−0.017	−0.013	0.01	−0.042	0.288*	0.037	0.502**	1	
DDAG	−0.049	−0.05	0.166	0.058	0.168	0.028	0.864**	0.531**	1

The significant correlations are indicated by **(*P* ≤ 0.01). PH: plant height (cm), NL: number of leaf, RL: root length (cm), TDW: total dry weight (g), proline (*μ*mol/g FW), TCE: total crude extract (g), AG: andrographolide (*μ*g/g dry weight), NAG: neoandrographolide (*μ*g/g dry weight), and DDAG: 14-deoxy-11,12-didehydroandrographolide (*μ*g/g dry weight).

**Table 6:** List of the 4 accessions of *Andrographis paniculata* collected from Peninsular Malaysia.

Number	Accession number	State	Vernacular name	Latitude	Longitude	Altitude (M)
1	11179	Selangor	Tutup Bumi	2° 56.454 N	101° 26.020 E	20
2	11264	Perak	Akar Cerita	5° 04.610 N	100° 23.561 E	−39
3	11266	Perak	Akar Cerita	5° 04.610 N	100° 23.561 E	−39
4	11329	Kelantan	Lidah Ular	3° 37.851 N	101° 02.759 E	−1

## Abbreviations

AC: Accession
AG: Andrographolide
DCM: Dichloromethane
DDAG: 14-Deoxy-11,12-didehydroandrographolide
ME: Methanol
NAG: Neoandrographolide
NL: Number of leaves
PH: Plant height
RL: Root length
SL: Salinity level
TCE: Total crude extract
TDW: Total dry weight.
